# Supplementary figures and images for: Timing of exposure is critical in a highly sensitive model of SARS-CoV-2 transmission
Source: PLoS Pathog. 2022 Mar 25;18(3):e1010181. doi: 10.1371/journal.ppat.1010181 (PMC8986102; doi:10.1371/journal.ppat.1010181)

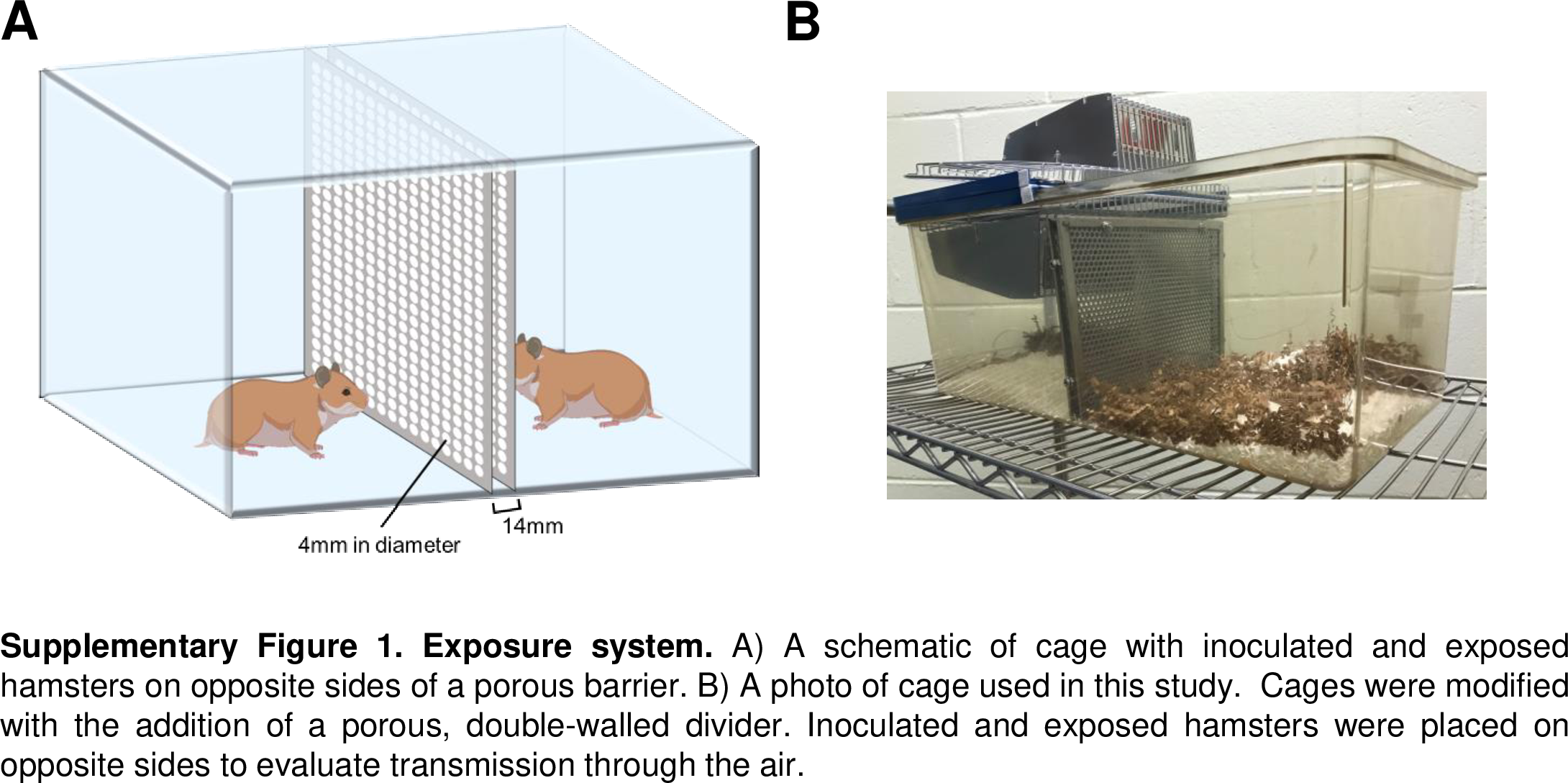

Supplement: S1 Fig — A] A schematic of cage with inoculated and exposed hamsters on opposite sides of a porous barrier. B] A photo of a representative cage used in this study. Cages were modified with the addition of a porous, double-walled divider. Inoculated and exposed hamsters were placed on opposite sides to evaluate transmission through the air. (TIF) [file ppat.1010181.s001.tif]

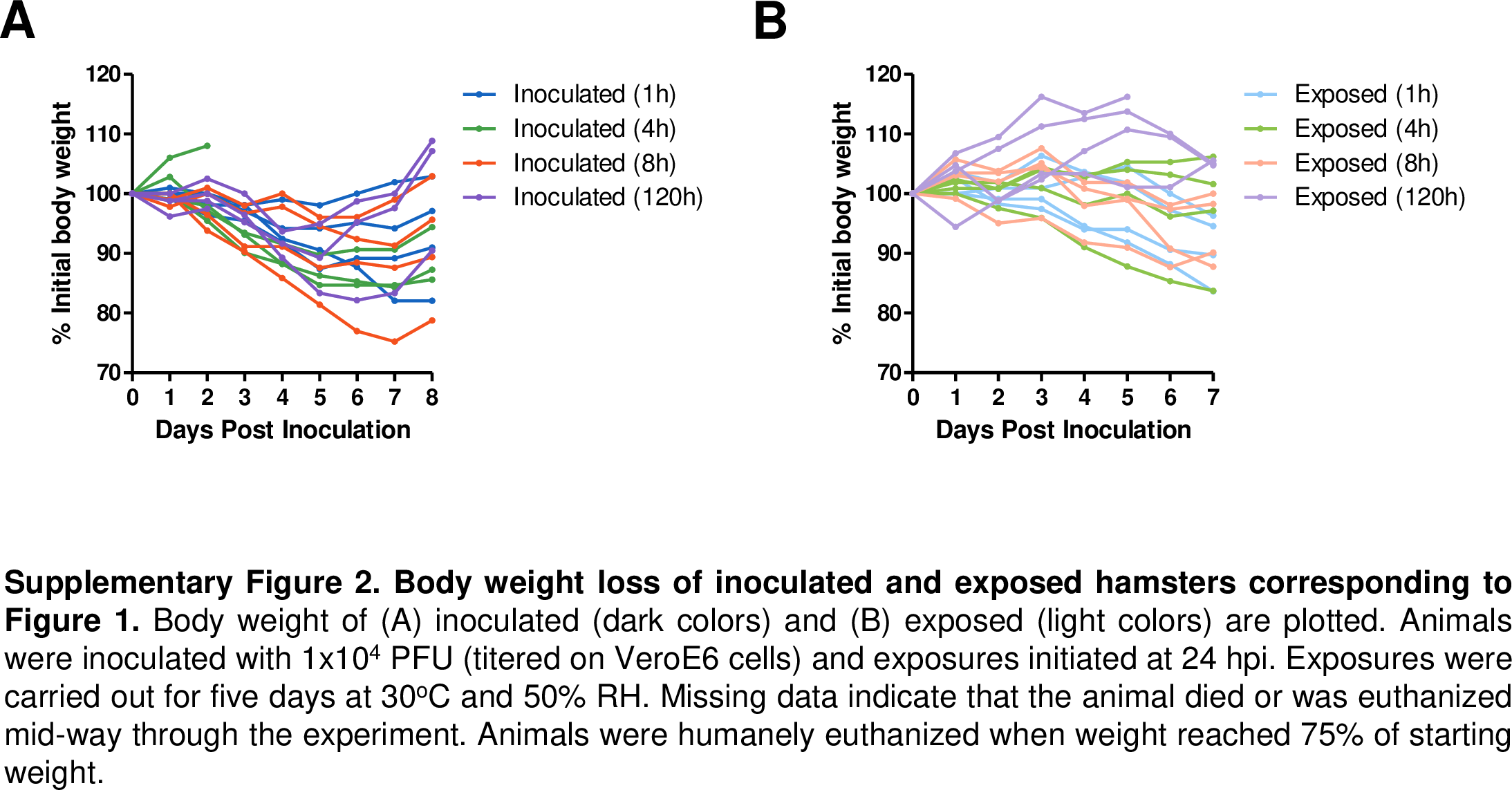

Supplement: S2 Fig — Body weights of [A] inoculated [dark colors] and [B] exposed [light colors] are plotted. Animals were inoculated with 1x10^4^ PFU [titered on VeroE6 cells] and exposures initiated at 24 hpi. Exposures were carried out for five days at 30°C and 50% RH. Missing data indicate that the animal died or was euthanized mid-way through the experiment. Animals were humanely euthanized when weight reached 75% of starting weight. (TIF) [file ppat.1010181.s002.tif]

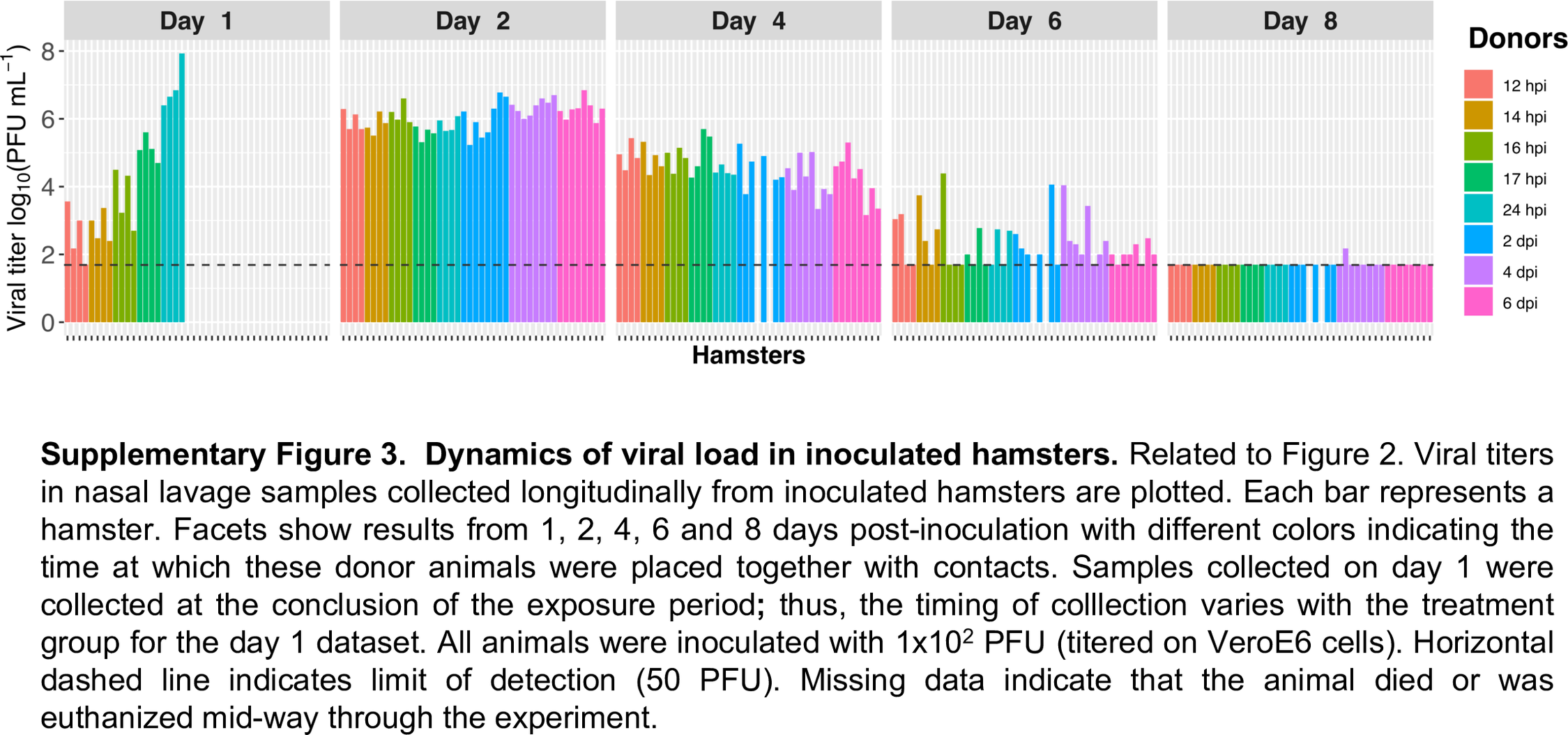

Supplement: S3 Fig — Related to Fig 2. Viral titers in nasal lavage samples collected longitudinally from inoculated hamsters are plotted. Each bar represents a hamster. Facets show results from 1-, 2-, 4-, 6- and 8-days post-inoculation with different colors indicating the time at which these donor animals were placed together with contacts. Samples collected on day 1 were collected at the conclusion of the exposure period; thus, the timing of collection varies with the treatment group for the day 1 dataset. All animals were inoculated with 1x10^2^ PFU [titered on VeroE6 cells]. Horizontal dashed line indicates limit of detection [50 PFU]. Missing data indicate that the animal died or was euthanized mid-way through the experiment. (TIFF) [file ppat.1010181.s003.tiff]

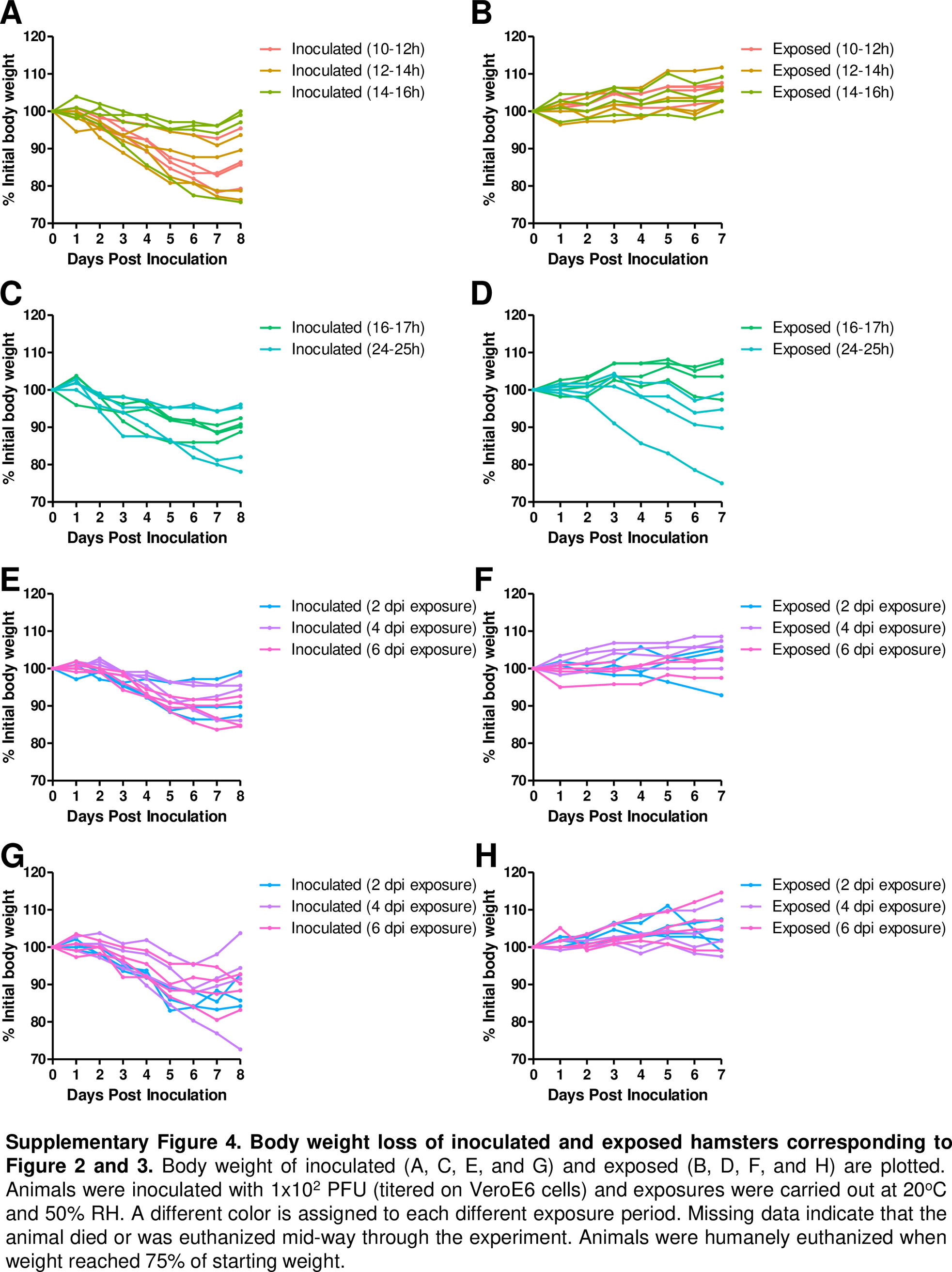

Supplement: S4 Fig — Body weights of inoculated [A, C, E, and G] and exposed [B, D, F, and H] are plotted. Animals were inoculated with 1x10^2^ PFU [titered on VeroE6 cells] and exposures were carried out at 20oC and 50%RH. A different color is assigned to each different exposure period. Missing data indicate that the animal died or was euthanized mid-way through the experiment. Animals were humanely euthanized when weight reached 75% of starting weight. (TIFF) [file ppat.1010181.s004.tiff]

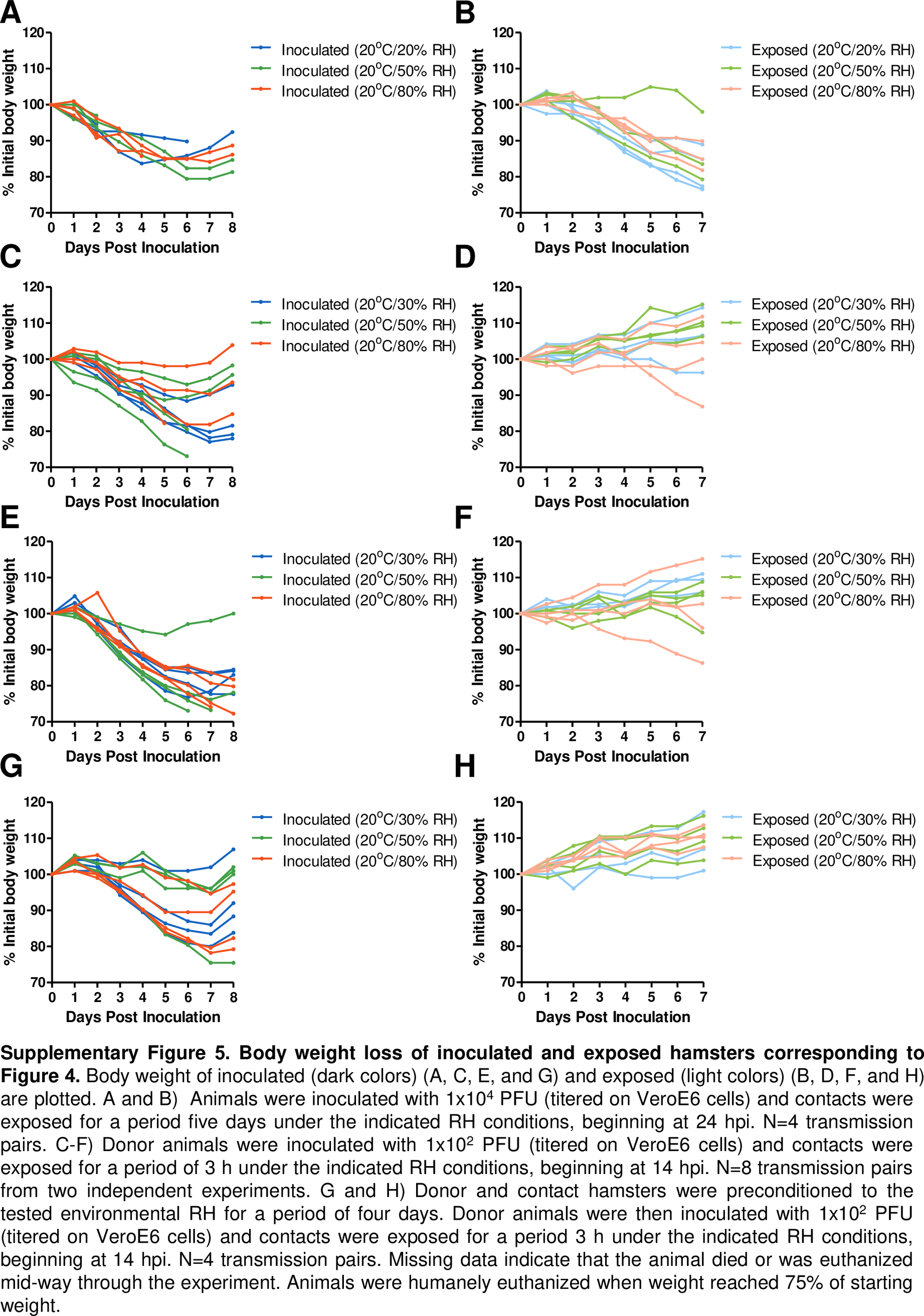

Supplement: S5 Fig — Body weights of inoculated [dark colors] [A, C, E, and G] and exposed [light colors] [B, D, F, and H] are plotted. A and B] Animals were inoculated with 1x10^4^ PFU [titered on VeroE6 cells] and contacts were exposed for a period of five days under the indicated RH conditions, beginning at 24 hpi. N = 4 transmission pairs. C-F] Donor animals were inoculated with 1x10^2^ PFU [titered on VeroE6 cells] and contacts were exposed for a period of 3 h under the indicated RH conditions, beginning at 14hpi. N = 8 transmission pairs from two independent experiments. G and H] Donor and contact hamsters were preconditioned to the tested environmental RH for a period of four days. Donor animals were then inoculated with 1x10^2^ PFU [titered on VeroE6 cells] and contacts were exposed for a period of 3h under the indicated RH conditions, beginning at 14 hpi. N = 4 transmission pairs. Missing data indicate that the animal died or was euthanized mid-way through the experiment. Animals were humanely euthanized when weight reached 75% of starting weight. (TIFF) [file ppat.1010181.s005.tiff]

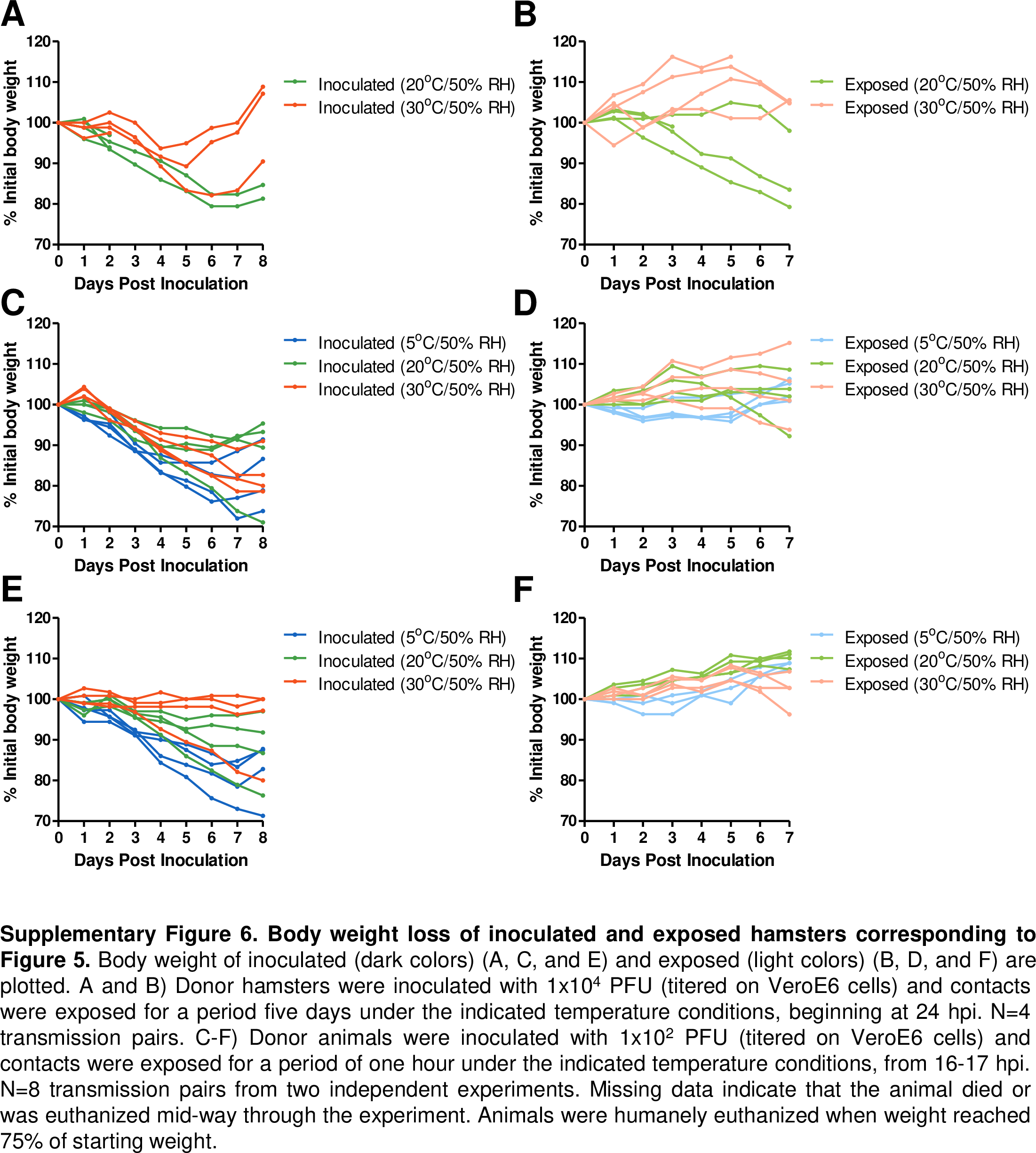

Supplement: S6 Fig — Body weight of inoculated [dark colors] [A, C, and E] and exposed [light colors] [B, D, and F] are plotted. A and B] Donor hamsters were inoculated with 1x10^4^ PFU [titered on VeroE6 cells] and contacts were exposed for a period of five days under the indicated temperature conditions, beginning at 24 hpi. N = 4 transmission pairs. C-F] Donor animals were inoculated with 1x10^2^ PFU [titered on VeroE6 cells] and contacts were exposed for a period of one hour under the indicated temperature conditions, from 16–17 hpi. N = 8 transmission pairs from two independent experiments. Missing data indicate that the animal died or was euthanized mid-way through the experiment. Animals were humanely euthanized when weight reached 75% of starting weight. (TIFF) [file ppat.1010181.s006.tiff]
