# Supplementary material for: Timing of exposure is critical in a highly sensitive model of SARS-CoV-2 transmission
Source: PLoS Pathog. 2022 Mar 25;18(3):e1010181. doi: 10.1371/journal.ppat.1010181 (PMC8986102; doi:10.1371/journal.ppat.1010181)
Supplement: Attachment — Submitted filename: Ganti timing of exposure on SARSCoV 2 PLoS Path 2022.docx [file ppat.1010181.s007.docx]

Ganti et al explore the effect of timing of exposure on SARS-CoV-2 transmission in a golden Syrian hamster model of SARS-CoV-2 transmission. As expected, exposure periods as brief as one hour are sufficient to support robust transmission. However, the timing after infection is critical for transmission success, with the highest frequency of transmission to contacts occurring at times of peak viral load in the donor animals. Humidity and temperature had no detectable impact on transmission when exposures were carried out at optimal timing. However, at sub-optimal exposure timing, transmission was improved at relatively higher levels of humidity or temperatures. While high humidity is logical for increased viral particle viability, the high temperature effects on transmission were not expected. The data presented is contradictory to the conclusions drawn in many places of the manuscript, making the manuscript confusing and many of the conclusions incorrect, in my opinion.

Major Concerns:

1. Regarding Figure 1, I completely disagree with the statement “Thus, within the range tested, the period of exposure had minimal impact on SARS-CoV-2 transmission in hamsters.” According to Figure 1, the animals clearly had more transmission events and resulted in higher viral titers when exposed with animals at days 4 or 6 post-inoculation as compared to animals exposed at days 2 or 8 post-inoculation. This needs to be quantified in several ways, including # transmission events, average viral titers of recipient animals, and perhaps a combination of both.
2. Donor animals loose their viral titer significantly after 2 days post-inoculation (dpi). In fact, according to supplementary Figure 2, the inoculated animals have their highest viral loads at 2 dpi. By 4 or 6 dpi, their viral titers are quite lower, and by day 8, the titers are basically zero. This seems to in complete contradiction with the donor animals having their best transmission times at 4 or 5 dpi, according to Figure 1. This is neither described or discussed, which is a major flaw of the manuscript.
3. There is clearly a discrepancy in the results from Fig. 2B-C and Fig. 1, when it comes to the timing of exposure post-donor inoculation. One possibility is that the viral loads are being determined in different ways for the two figures. The most accurate method should be plaque assays. Where viral loads determined via plaque assays for both experiments? If not they should be. Also, these discrepancies need to be explained experimentally and in the Results/Discussion sections. They are very obvious and largely ignored.
4. In Figure 3, both 16-17 and 48-50 hpi are shown as red dots. Times of exposure 0-12 hrs and 12-14 hours are also the same colors, so it is impossible to distinguish between them. Therefore, it is impossible to interpret this figure.
5. I completely disagree with this conclusion regarding the humidity studies in Fig. 4: “These results clearly indicated that, contrary to expectation, low ambient humidity was not favorable for transmission.” I think the field out there agrees fairly well than lower humidity does not favor transmission, while high humidity does.
6. I am not convinced on the temperature results in Figure 5 either, given that the animals were at these temperatures (it seems), which will affect their behavior, movement, etc… and these factors were not controlled.
7. The system used may not as natural as allowing the animals to naturally interact. It does have the value of having a more control exposure experimental system, but it is more artificial as far as how animals would naturally interact. This should at least be discussed in the manuscript.
8. No information for the size of the pores of the walls between chambers is included in the manuscript. This is very important to calculate whether all types of aerosol particles can travel through the walls.
