# Supplementary material for: Timing of exposure is critical in a highly sensitive model of SARS-CoV-2 transmission
Source: PLoS Pathog. 2022 Mar 25;18(3):e1010181. doi: 10.1371/journal.ppat.1010181 (PMC8986102; doi:10.1371/journal.ppat.1010181)
Supplement: Attachment — Submitted filename: Response to Reviewers_transmission of SARS2_R1 Final.docx [file ppat.1010181.s008.docx]

Dear Dr Jurado,

We are happy to submit a revised version of our manuscript entitled “Timing of exposure is critical in a highly sensitive model of SARS-CoV-2 transmission.” (PPATHOGENS-D-21-02474R1). We have reproduced the reviewers' comments on the following pages and detailed our responses underneath in blue font. The modifications made include the addition of clarifications within the text. The reviewers provided insightful comments and we would like to thank them for improving the quality of this work.

Sincerely,

Ketaki Ganti and Anice Lowen

**Response to Reviewers**

**Reviewer #1**

**Part I - Summary**

In this manuscript, Ganti et al. evaluate how timing of exposure, exposure duration, as well as humidity and temperature impact SARS-CoV-2 transmission in highly controlled experimental settings, using a hamster model of infection.
While the authors found that exposure duration as short as 1h was sufficient to enable viral transmission, the timing of exposure appeared as a critical parameter for successful viral transmission. Especially, authors found that successful viral transmission associated with a time when viral load peaks in the nasal cavity of the animals, that is between 17h to 48h post infection. Finally, they addressed the role of humidity and temperature on viral transmission and suggest that low temperature and humidity prevent effective transmission of SARS-CoV-2. This work contributes to fill up an important knowledge gap and bring new lights as to the roles of viral and environmental parameters on SARS-CoV-2 transmission dynamics. Clearly, the value of this work lies within the fact that authors analyze the impact of these different parameters on viral transmission using a well characterized animal model of SARS-CoV-2 infection and highly controlled experimental settings. However, while this work provides increased clarity as to the relative impact of such parameters on transmission, a fundamental question is whether the reported findings are specific to the model and experimental settings used in this study or can be directly relatable to human-to-human transmission.

We thank the reviewer for their supportive comments. We have addressed the concern about translation of the information to human settings below in response to point 3.

**Part II – Major Issues: Key Experiments Required for Acceptance**

1.Despite being rather expected and hardly serving the novelty potential of the study, the major take-away of this work – that the peak of viral load associate with effective transmission – is likely translatable to human transmission and represent as such a comprehensive confirmation of an association between high viral load and high risk of transmission. In contrast, how the subjacent findings related to exposure timing can be harnessed to increase our understanding of human-to-human SARS-CoV-2 transmission remain uncertain (and honestly stated by the authors, line 265) given the specificities of the experimental system and parameters in use in this study. Most importantly, experiments assessing the impact of different temperature and humidity are relatively confusing. Indeed, as transmission efficiency appears to be dependent of time of exposure and incubation period under similar RH or temperature conditions, the translational nature of these findings and how they may increase our understanding of SARS-CoV-2 transmission between humans is unclear. This is emphasized by evidence that the RH and temperature findings in this study are in contradiction with previous reports, and by the fact that authors admit that their unexpected results may be driven by the specific experimental settings they are using.

We agree with the reviewer that our finding regarding the effects of humidity and temperature on transmission were unexpected. This issue is discussed at lines 254-276 and is reproduced below:

“These observations led to our hypothesis that SARS-CoV-2 transmission would be suppressed by high ambient humidity and temperature conditions. Our data do not validate this hypothesis and instead reveal efficient transmission among hamsters housed in humid or warm environments. Robust transmission under humid conditions is consistent with the U-shaped relationship between RH and stability that has been reported for multiple enveloped viruses, including SARS-CoV-2 (48-50). In general, however, these results were unexpected based on reports of super-spreading events in cold or dry indoor environments (51-54) and available information on how temperature and RH modulate viral stability, host susceptibility and aerosol dynamics (50, 55-57). The unexpected results may stem from our approach of performing exposures early in the course of infection; transmission success may be more subject to stochastic effects at early times compared to exposures carried out at times when viral load in donors is at peak levels.

While circulation of endemic coronaviruses shows a clear seasonal pattern (58-60), increased activity in winter has not been a consistent feature of the COVID-19 pandemic. Influenza pandemics also do not follow the typical seasonality of epidemic influenza but have shown a decline in circulation during summer months (61, 62), suggesting the relative influence of seasonal drivers is reduced but not ablated for pandemic influenza. In the case of SARS-CoV-2, a lack of seasonal patterns at this early stage in its circulation may reflect low population immunity, dominance of other epidemiological factors such as population behavior and government interventions (16), or a lack of sensitivity to the seasonal drivers that shape the dynamics of epidemic coronaviruses and seasonal influenza viruses. While our data suggest a lack of sensitivity to high humidity and temperature, we caution against over-interpretation of these data given the limitations inherent in our experimental system.”

2.Of note, it is unclear to me why the authors used a 1e4 PFU viral inoculum for assessing the impact of high/low temperature and RH in optimal transmission settings (Figure 4A and 5A; 5 days exposure, 24hpi) while using a 1e2 PFU viral dose in the context of the sub-optimal transmission settings (Figure 4B,C and 5B) although they mention in the main text that the experiments between optimal and suboptimal environmental settings were otherwise conducted similarly (“similar” line 177 and 218). It remains unclear to me why authors decided not to use the same viral dose across all experiments, i.e., the one they used in earlier experiments (1e2 PFU) when setting optimal transmission settings. One therefore cannot exclude that a viral dose of 1e4 in Figure 4A and 5A (a dose that is not used when assessing timing of exposure and exposure duration) could have prevented to observe any environmental impact on viral transmission, because it would have been too high of a dose.

We thank the reviewer for their comment. Dose was changed mid-way through our experiments as part of our effort to identify experimental conditions in which transmission was not saturated. We agree that, since the effect of dose is not explicitly tested, we need to be clearer about how dose varies with experiment. To this end, we have modified the text at lines 27-30; 160-165 and 189-198, to state that both timing of exposure and dose were changed to achieve ‘sub-optimal’ transmission conditions. These changes are reproduced below (see underlines):

“Relative humidity and temperature had no detectable impact on transmission when exposures were carried out with optimal timing and high inoculation dose. However, contrary to expectation, trends observed with sub-optimal exposure timing and lower inoculation dose suggest improved transmission at high relative humidity or high temperature.”

“When a high inoculation dose and a five-day exposure duration beginning at 24 hpi were used, transmission was found to be highly efficient irrespective of humidity (**Figure 4B**). Reasoning that any effects of humidity may be difficult to detect when overall transmission efficiencies are saturated, we performed similar experiments with a reduced inoculation dose and exposures carried out early after infection when intermediate transmission frequency was observed.”

“When a five-day exposure duration beginning at 24 hpi was used to compare 20^o^C and 30^o^C environments, transmission was found to be highly efficient under both conditions (**Figure 5B**). Since our hypothesis was that cold conditions would be favorable, we did not test 5^o^C in this system. Instead, we performed similar experiments with a lower inoculation dose and a one-hour exposure beginning at 16 hpi – an approach designed to yield intermediate levels of transmission and thereby allow detection of temperature effects. A schematic of the transmission experimental setup is shown in **Figure 5C**. While intermediate levels of transmission were observed at 20^o^C and 30^o^C in these experiments, no transmission was observed at 5^o^C. Thus, results did not support the hypothesis that cold conditions augment transmission of SARS-CoV-2 **(Figure 5D).**”

3. Overall, beyond the uncertainty regarding the translational nature of this study’ findings to human transmission, the experimental parameters defining viral transmission under specific environmental settings with the hamster model itself also remain unclear.

We have added further discussion regarding features of the study that may be translatable to humans, as follows (lines 221-233):

“Clinical data suggest that peak viral loads occur 3–4 days after infection (35, 36) and results of an experimental human challenge study similarly show peak loads in the throat at day 4 and in the nose at day 5 post-infection (37). In our experiments, peak titers were seen in hamsters much earlier, at 24 hpi. This difference of kinetics may relate to initial dose, as these doses of 1x10^2^–1x10^4^ PFU used here likely exceed a typical natural dose and are higher than that used in the human challenge study. Indeed, examination of viral titers in recipient animals shows delayed kinetics of replication for brief, early exposures – likely associated with lower initial dose – compared to exposures carried out over an extended period or at the peak of donor viral load. As a result, the period of contagiousness identified in hamsters is unlikely to translate directly to human hosts. However, the observation that the period of contagiousness corresponds to times of high viral load is likely to extend to humans. Comparing our data to those from experimentally infected humans (37), we would therefore infer that the period of peak contagiousness in humans is between approximately days 3 and 8 post-infection.”

**Part III – Minor Issues: Editorial and Data Presentation Modifications**

1. As authors established that 5°C exposure prevents transmission under sub-optimal transmission settings, authors should test this temperature under optimal condition to strengthen evidence for its potential impact on transmission.

We thank the reviewer for this comment. We respectfully disagree that we have “established that 5°C exposure prevents transmission”. As we state in the Results section, we conclude that our results from transmission experiments performed at this low temperature “did not support the hypothesis that cold conditions augment transmission of SARS-CoV-2”. We feel that it is clear from the data currently presented that results obtained in the hamster model do not substantiate the effects of RH and temperature that would be expected based on coronavirus seasonality. While we appreciate the suggestion, we therefore do not wish to invest the substantial resources needed to perform the suggested experiment.

2. Can the authors provide any clinical information from the donor and exposed animals? Were (all) the transmission events inducing weight loss?

We have included graphs showing daily body weight data for both donor and exposed animals under all conditions tested in Supplementary Figures 2, 4-6. Text has been added to each results section to briefly describe these results. For example, at lines 113-117 as reproduced here:

“In inoculated animals, body weight loss was typically moderate to severe. While results were more variable for contact animals, the timing of their weight loss usually corresponded to the timing with which infectious virus was first detected in nasal washes (**Supplementary Figure 2**). Occasionally, infected hamsters lost greater than 25% of their initial body weight and were euthanized.”

3. Line 134: Should this write “12, 14 or 16h”?

We have made the change.

4. Line 135-136: Should this write “beginning at 17h”?

We have made the change.

**Reviewer #2**

**Part I - Summary**

The manuscript by Ganti et al. describes the effects of exposure period, timing, and coincident variables such as relative humidity and ambient temperature on the transmission efficiency of SARS-CoV-2, which was measured by measuring viral load in the nasal washes of Syrian hamsters co-housed (1:1) with infected “donor” hamsters on opposite sides of a porous barrier. The authors first show that exposure periods as short as 1 hour within this system are sufficient for transmission, when the donor hamsters were infected 24 hours prior to exposure of the recipient, or “contact”, hamsters. Next, the authors varied the time post-infection when infected donor hamsters were co-housed with contact hamsters, finding that donor hamsters co-housed in a window of 16 hours – 2 days post-infection were the most likely to transmit. This period also correlated with highest viral load in donor hamsters. Congruously, the authors found that the viral loads of donor hamsters positively correlated with whether contact animals became infected or not. Lastly, the authors varied two environmental variables, relative humidity and temperature, and found that high relative humidity and high temperature each tended to promote SARS-CoV-2 transmission. Overall, the work credibly evaluates the relationships between transmission of SARS-CoV-2 and exposure timing, viral load of donor individuals, relative humidity, and temperature. Exposure timing corresponding with viral load drives the greatest amount of SARS-CoV-2 transmission. While somewhat expected, this finding confirms that virus load and the timing of peak virus titer is important for transmission. The authors could better link this to peak viral load in humans in the discussion. Similarly, the evaluation of humidity and temperature provide an unexpected result. Both higher humidity and higher temperature were associated with increased transmission, contrasting what was expected based on influenza studies. While the result is clear for both temperature and humidity, it would be beneficial for the authors to speculate a potential mechanism for these differences. Together, the findings shed light on variables affecting SARS-CoV-2 transmission, highlighting the central importance of viral load to transmission.

We thank the reviewer for their thoughtful comments. We have included text in the discussion to link our observations to transmission in humans at lines 214-233. We have also included points in the discussion about potential mechanisms for the effects of temperature and humidity on transmission at lines 248-265.

**Part II – Major Issues: Key Experiments Required for Acceptance**

No additional experiments required

**Part III – Minor Issues: Editorial and Data Presentation Modifications**

1. A schematic of the transmission experiments (number of animals co-housed) would be beneficial in the main text or in the supplementary text. At a minimum, please state the number of animals (1:1) in the figure legend.

We thank the reviewer for their suggestion. We have included a schematic of the 1:1 transmission set up in Supplementary Figure 1 and added a timeline to each figure that shows how the transmission experiments were conducted.

2. In figure 1, while the legends are reasonable, it would be helpful to label the times on the X axis for clarity. There is a significant amount of data/information and cross referencing to the legend is a bit tedious if labels can be added.

We appreciate the feedback. To improve clarity, we have added a schematic timeline to each figure that shows how the transmission experiments were conducted. The timeline includes time points to show timing and duration of exposure.

3. Supplemental figure 2 could be included in main text. Supplemental figure 1 could be removed.
We have removed the original Supplemental Figure 1 as suggested and replaced it with the requested schematic showing the 1:1 transmission design. This figure also retains an image of the cages used as these constitute an important feature of the experimental design. We have elected to keep Supplemental Figure 2 (now Supplemental Figure 3) in the supplement, as we fear that adding it to the relevant main figure would make it very confusing. The focus of this figure is on the timing of exposure, making the addition of kinetic data to show detailed results of viral shedding, not transmission, problematic.

4. Ideally, a time point at 36 hours would have been helpful. However, the results are clear that peak viral titer (25hr-2DPI) associate with the most robust transmission period. The authors should comment on if a minimum amount of virus is necessary for transmission. Specifically observing differences in transmission at 17HPI and 4 days though titers are similar.

We thank the reviewer for this suggestion. We have added information to the Discussion to address this comment, including a comparison of our results to those obtained in a recently reported experimental human challenge study (lines 221-233 and reproduced below):

“Clinical data suggest that peak viral loads occur 3–4 days after infection (35, 36) and results of an experimental human challenge study similarly show peak loads in the throat at day 4 and in the nose at day 5 post-infection (37). In our experiments, peak titers were seen in hamsters much earlier, at 24 hpi. This difference of kinetics may relate to initial dose, as these doses of 1x10^2^–1x10^4^ PFU used here likely exceed a typical natural dose and are higher than that used in the human challenge study. Indeed, examination of viral titers in recipient animals shows delayed kinetics of replication for brief, early exposures – likely associated with lower initial dose – compared to exposures carried out over an extended period or at the peak of donor viral load. As a result, the period of contagiousness identified in hamsters is unlikely to translate directly to human hosts. However, the observation that the period of contagiousness corresponds to times of high viral load is likely to extend to humans. Comparing our data to those from experimentally infected humans (37), we would therefore infer that the period of peak contagiousness in humans is between approximately days 3 and 8 post-infection.”

5. The colors in figure 3 need to be modified to be more distinct, especially 16-17 vs 48-50 as the colors cannot be discerned as constructed. With that said, the trend lines are convincing.

We thank the reviewer for spotting the inconsistency. We have modified Figure 3 to make the colors more discernable.

6. Figures 4 and 5 could provide additional labels or would benefit from a schematic. It is difficult to decipher exactly what is going on in the figure from the legend. This can be included in the main text or supplement.

We thank the reviewer for their suggestion. We have included a schematic for how the transmission experiments were conducted in each figure.

7. In the sentence beginning on Line 124, it was unclear whether the timepoints given (e.g., in Line 124) refer to the time when contact animals were co-housed with donor animals or the time when donor animals were sampled for their own viral loads. The sentence beginning Line 127 should be similarly edited for clarity.

We thank the reviewer for spotting these issues. We have corrected the text, now at lines 120-126, as shown below:

“To test for transmission at early times, brief exposures of one or two hours were carried out at 10–12, 12–14, 14–16, 16–17 or 24–25 hpi. Similarly, to evaluate transmission potential late in the course of infection, exposures were carried out for two hours on days 2, 4 and 6 post-inoculation. Nasal lavage samples collected from donor animals at the conclusion of each exposure window were used to assess viral loads at the time of exposure, while serial samples collected from these same donors across multiple time points were used to evaluate the dynamics of viral load.”

8. Figure 3 lacks a line delineating the limit of detection.

We have made the correction.

9. The phrase in Line 272: "transmission efficiency declines with infectious viral titers" is worded awkwardly and seems to contradict the data.

We changed the phrasing at line 238 to “the frequency of transmission declines with viral titers”. This conclusion is drawn from the data shown in Figure 2 and is consistent with those results.

10. There is a lack of statistical testing in much of the text. A test to compare proportions of infected animals could perhaps add additional rigor.

We have included the results of Fisher’s exact tests to compare proportions of infected animals under differing exposure conditions. The p-values are indicated in the figure legends.

**Reviewer #3**

**Part I - Summary**

Ganti et al. explore the effect of timing of exposure on SARS-CoV-2 transmission in a golden Syrian hamster model of SARS-CoV-2 transmission. As expected, exposure periods as brief as one hour are sufficient to support robust transmission. However, the timing after infection is critical for transmission success, with the highest frequency of transmission to contacts occurring at times of peak viral load in the donor animals. Humidity and temperature had no detectable impact on transmission when exposures were carried out at optimal timing. However, at sub-optimal exposure timing, transmission was improved at relatively higher levels of humidity or temperatures. While high humidity is logical for increased viral particle viability, the high temperature effects on transmission were not expected. The data presented is contradictory to the conclusions drawn in many places of the manuscript, making the manuscript confusing and many of the conclusions incorrect, in my opinion.

**Part II – Major Issues: Key Experiments Required for Acceptance**

1. Regarding Figure 1, I completely disagree with the statement “Thus, within the range tested, the period of exposure had minimal impact on SARS-CoV-2 transmission in hamsters.” According to Figure 1, the animals clearly had more transmission events and resulted in higher viral titers when exposed with animals at days 4 or 6 post-inoculation as compared to animals exposed at days 2 or 8 post-inoculation. This needs to be quantified in several ways, including # transmission events, average viral titers of recipient animals, and perhaps a combination of both.

We respectfully disagree with the reviewer’s conclusion since we believe the results have been misinterpreted. To avoid this issue going forward, we have included a schematic of the experimental design in Fig 1 for clarity. Briefly, animals were exposed to inoculated donors beginning at 24hpi for various durations (1h, 4h, 8h or 120h). Nasal washes were collected at Days 2, 4, 6 and 8 from both inoculated and exposed animals and analyzed for viral shedding. We have included a quantification of the number of transmission events in Fig 1, which show 100% transmission under all conditions tested, indicating that the period of exposure had no detectable impact on transmission.

2. Donor animals lose their viral titer significantly after 2 days post-inoculation (dpi). In fact, according to supplementary Figure 2, the inoculated animals have their highest viral loads at 2 dpi. By 4 or 6 dpi, their viral titers are quite lower, and by day 8, the titers are basically zero. This seems to in complete contradiction with the donor animals having their best transmission times at 4 or 5 dpi, according to Figure 1. This is neither described nor discussed, which is a major flaw of the manuscript.

Please refer to the response above. We believe the reviewer may have mis-interpreted the figure. In Fig 1, the exposed animals do show peak shedding at Day 4 or 6, but these animals were exposed to the donors at 24hpi, when viral shedding from the donor animals is at its peak.

3. There is clearly a discrepancy in the results from Fig. 2B-C and Fig. 1, when it comes to the timing of exposure post-donor inoculation. One possibility is that the viral loads are being determined in different ways for the two figures. The most accurate method should be plaque assays. Where viral loads determined via plaque assays for both experiments? If not they should be. Also, these discrepancies need to be explained experimentally and, in the Results/Discussion sections. They are very obvious and largely ignored.

We believe that the reviewer may be misinterpreting the data shown in Fig 1 and 2, as the differing experimental outcomes result from differing times of exposure. In Fig 1, all animals were exposed at 24hpi of donors for varying lengths of time as now depicted in the schematic in Fig 1A. In Fig 2, animals were exposed at different time points post infection of donors as now shown in the schematic in Fig 2A. Exposure of these animals was performed for a period or 1h or 2h. Viral loads for all experiments were determined using plaque assay. We hope that the addition of the schematics will alleviate any further confusion regarding the experimental setup and interpretation of data.

4. In Figure 3, both 16-17 and 48-50 hpi are shown as red dots. Times of exposure 0-12 hrs and 12-14 hours are also the same colors, so it is impossible to distinguish between them. Therefore, it is impossible to interpret this figure.

We thank the reviewer for catching this error. We have modified Figure 3 to more clearly depict the times of exposure.

5. I completely disagree with this conclusion regarding the humidity studies in Fig. 4: “These results clearly indicated that, contrary to expectation, low ambient humidity was not favorable for transmission.” I think the field out there agrees fairly well than lower humidity does not favor transmission, while high humidity does.

We respectfully disagree with the reviewer’s comment about the field at large agreeing that lower humidity does not favor transmission. The most comprehensive experimental studies on the impact of humidity on transmission of respiratory viruses has been done using influenza A viruses in guinea pigs (Lowen et. al 2007, 2008), which clearly show that low humidity is favorable for aerosol transmission. We are not aware of any experimental studies using SARS-CoV-2 which investigate the impact of humidity on transmission.

6. I am not convinced on the temperature results in Figure 5 either, given that the animals were at these temperatures (it seems), which will affect their behavior, movement, etc… and these factors were not controlled.

The animals were monitored daily in consultation with the clinical veterinarian as dictated by our IACUC protocol. The animals were housed at the cold temperatures for 1h, which was not found to significantly impact their behavior. We have included text in the methods at lines 310-312 and is reproduced below:

“Animal health was monitored daily through visual observation and determination of body weight in consultation with the clinical veterinarian as dictated by the IACUC protocol.”

7. The system used may not as natural as allowing the animals to naturally interact. It does have the value of having a more control exposure experimental system, but it is more artificial as far as how animals would naturally interact. This should at least be discussed in the manuscript.

While we agree with the reviewer that our exposure system would benefit from allowing natural interactions between the animals, male hamsters kept in pairs tend to fight and can cause stress and injury. In consultation with veterinarians and IACUC, we therefore decided to keep the animals housed individually to avoid any stressful situations for the animals. We have included text in the methods at lines 306-307 and is reproduced below:

“The hamsters were singly housed to avoid stress for the animals since male hamsters kept in pairs tend to fight and can cause injury.”

8. No information for the size of the pores of the walls between chambers is included in the manuscript. This is very important to calculate whether all types of aerosol particles can travel through the walls.

We agree with the reviewer and have included the information in Supplementary Fig 1 as well as at lines 328-331 and is reproduced below:

“Exposures were carried out in rodent cages modified through the addition of a double-walled porous barrier, which divided the cage in two. A single hamster was placed on either side of the barrier. The barrier reached from wall-to-wall and floor-to-lid and comprised two stainless-steel sheets placed 14 mm apart, with perforations 4 mm in diameter arrayed across each sheet”
